# Editorial: Assessing sleep neuroplasticity in pathological conditions and in extreme environments through neurophysiological and multi-faceted daily lifestyle patterns

**DOI:** 10.3389/fnins.2023.1216794

**Published:** 2023-05-22

**Authors:** Chrysoula Kourtidou-Papadeli, Christiane M. Nday, Myrto Samara, Christos A. Frantzidis

**Affiliations:** ^1^Laboratory of Medical Physics and Digital Innovation, Biomedical Engineering and Aerospace Neuroscience (BEAN), School of Medicine, Faculty of Health Sciences, Aristotle University of Thessaloniki, Thessaloniki, Greece; ^2^Greek Aerospace Medical Association and Space Research (GASMA-SR), Thessaloniki, Greece; ^3^Aeromedical Center of Thessaloniki (AeMC), Thessaloniki, Greece; ^4^Department of Psychiatry, Faculty of Medicine, University of Thessaly, Larisa, Greece; ^5^School of Computer Science, University of Lincoln, Lincoln, United Kingdom

**Keywords:** sleep, extreme environment, spaceflight adaptation, brain mapping, neuroplasticity and exercise, Short Arm Human Centrifuge, isolation, confinement

This article collection aimed to highlight the crucial role of sleep during human exploration of outer space and extreme environments in general. Astronauts and cosmonauts need to preserve optimal physical and mental wellbeing to avoid cognitive derangement and performance decrement. We invited contributions that would investigate how sleep quality is affected by extreme environments as well as mitigation approaches. Therefore, our objective was to identify a roadmap toward long-term space spaceflights and Mars missions.

The systematic review of Zivi et al., investigated the sleep quality in several analog environments aiming to simulate the isolated, confined, and extreme conditions occurring during spaceflights. Although most of the findings focus on deep sleep degradation, the authors re-assessed the existence of complex and often contradictory patterns of sleep deterioration. This contradiction stems from the multifactorial set of environmental and biological stressors that impede sleep. Another major issue is that the highly demanding experimental conditions limit the sample size and increase the variability of the findings.

A significant contribution by Wong et al. presented the research version of a NASA's platform, the Robotics On-Board Trainer (ROBoT-r), which is a training task used to improve the docking and grappling maneuvers of the astronauts. The authors performed a sleep deprivation study for 28 h in laboratory settings and investigated whether the astronauts' performance was affected. They identified that continued learning resulted in overall improved performance despite the sleep loss. However, their psychomotor vigilance was reduced. The importance of this study is that is shows that highly skilled individuals recruit compensatory mechanisms to maintain their optimal working performance on complex, novel tasks for a given temporal window. The authors acknowledge that a platform update with secondary tasks is needed to shed light into the vulnerabilities caused by sleep deprivations. To that point an individual data analysis in needed after reaching task proficiency.

Apart from the unique conditions occurring in the International Space Station and spaceflights, the pandemic crisis offered another major experimental testbed for sleep quality assessment. There was a major public concern regarding the vaccination program to control the COVID-19 outbreak. Vaccination coverage was limited in many cases due to adverse reactions (ARs) concerns. Xiao et al. investigated through a longitudinal study, whether the sleep quality of healthcare workers is degraded after vaccination and if there is a relationship among sleep quality and AR incidence. The authors reported a bidirectional relationship formulated as a statistically significant deterioration in sleep quality after vaccination, while poor sleepers were more prune to ARs in comparison with good sleepers.

Similarly with the spaceflights, the COVID-19 associated, confinement policies induced stress levels and situational insomnia. These specific conditions motivated researchers to establish digital interventions administered as self-guided treatments. Within that context, Zhang et al., investigated the efficacy of 1-week Internet cognitive behavioral treatment for situational insomnia (CBTI) in 184 individuals assigned either to a training or a control group. The outcome measures indicate that the intervention could alleviate insomnia symptomatology and treat pre-sleep somatic hyperarousal. The treatment of insomnia is of crucial importance to avoid driver accidents due to mind wandering (MW) performance. Xu et al. investigated whether 21 insomnia patients show diminished driving performance in comparison with an equal size of healthy controls. The authors employed a driving simulator experiment involving both a no-distraction and MW task. The study findings indicate that the combination of the insomnia with a cognitively challenging MW task can degrade the driving performance.

Another major scientific domain is the association of sleep quality and cognitive functioning. Li et al. recruited 24 healthy college students who completed mental rotation tasks before and after total sleep deprivation (TSD). Apart from the task accuracy, the authors estimated the P300 amplitude, its localization, and the underlying effective connectivity among the cortical generators of that Event-Related-Potential (ERP) component. The study findings highlighted the detrimental TSD impact on cognition, which is also reflected in smaller P300 amplitude and elevated effective connectivity between the middle frontal gyrus and parietal cortices. Similar results were also reported by Song et al. regarding motor preparation processing in a visual search task.

This article collection highlighted the crucial role of sleep in preserving an optimal cognitive performance either on earth or in long-term spaceflights. However, there is still a lot of work that should be undertaken to (a) provide concrete evidence on sleep degradation in space (b) mitigate these disturbances through cost-effective interventions. Future perspectives on sleep physiology in space medicine and clinical practice may include an integrative assessment of sleep. A proposed roadmap that will enable optimal cognitive performance and sleep quality is shown in Figure 1. It includes electroencephalographic (EEG) recordings in resting state (Frantzidis et al., 2014) and during the performance of cognitive tasks. These would be fused with polysomnographic (PSG) recordings to extract neurocognitive markers able to detect sleep disorders (Chriskos et al., 2019) at their onset (Frantzidis et al., 2020). Then, personalized interventions through Short Arm Human Centrifuge (SAHC) could provide a robust countermeasure through a combination of artificial gravity combined with aerobic and resistance training (Kourtidou-Papadeli et al., 2021).

**Figure 1:**
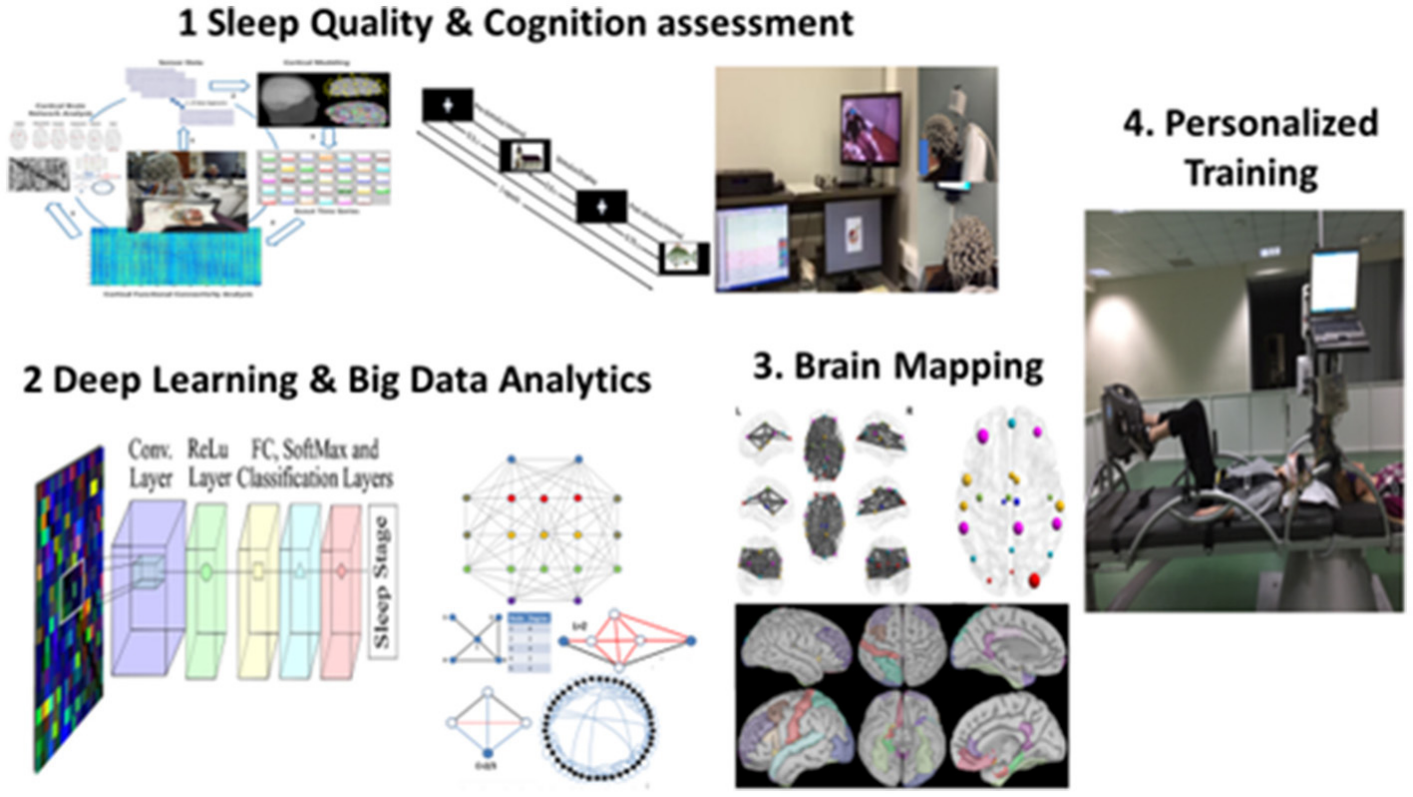
Concept of an integrative assessment of sleep quality and cognitive functioning through multi-modal recordings. The big data collection leads to the application of deep learning techniques that could be fused with brain mapping methodologies to provide markers of sleep degradation. These neurocognitive markers could be then used to formulate personalized protocols through multi-system artificial gravity and physical activity training.

## Author contributions

All authors contributed to this article and accepted the final version.
